# Safety and Efficacy of Stem Cell Therapy in Patients With Ischemic Stroke

**DOI:** 10.7759/cureus.9917

**Published:** 2020-08-21

**Authors:** Jeevan Gautam, Amer Alaref, Abdallah Hassan, Rajan Sharma Kandel, Rohi Mishra, Nusrat Jahan

**Affiliations:** 1 Neurology, California Instititute of Behavioral Neurosciences and Psychology, Fairfield, USA; 2 Diagnostic Radiology, California institute of Behavioural Neurosciences and Psychology, Fairfield, USA; 3 Diagnostic Radiology, Thunder Bay Regional Health Sciences Centre, Thunder Bay, CAN; 4 Diagnostic Imaging, Northern Ontario School of Medicine, Sudbury, CAN; 5 Breast Imaging Services, Linda Buchan Centre, Thunder Bay, CAN; 6 Internal Medicine, California Institute of Behavioral Neurosciences & Psychology, Fairfield, USA

**Keywords:** stem cells, ischemic stroke, clinical trials, stroke treatment, stem cell therapy

## Abstract

Stem cell therapy is emerging as a promising treatment strategy to treat patients with stroke. While there are established modes of treatment for stroke patients such as thrombolysis and endovascular intervention, most of the stroke patients frequently end up with major residual deficits or even death. The use of stem cells to treat stroke has been found to be beneficial in the animal models but strict evidence for the same in humans is still lacking. We reviewed 13 clinical trials of stem cell therapy in stroke patients conducted between 2014 and 2020 based on the search using the database PubMed, and the clinical trial registry (www.clinicaltrials.gov). We aimed to assess the safety and efficacy of stem cell treatment in stroke patients who participated in the trials. Quality assessment of the clinical trials revealed a sub-optimal score. We found mixed results regarding the efficacy of stem cells in the treatment of ischemic stroke although we could not do a quantitative analysis of the effect outcomes. Assessment for safety revealed promising results as there were only minor side effects related to cell therapy. Although stem cell therapy seems to be a promising strategy to treat stroke patients in the future, we concluded that the field needs more evidence regarding the safety and efficacy of the use of stem cells in stroke patients before we use them in the clinic.

## Introduction and background

Stroke is the second largest cause of death globally (~5.5 million) and also the second most common cause of global disability-adjusted life years (~116.4 million) [1,2]. Stroke prevalence in adults is 2.9% in the United States, with about 795,000 people experiencing a new or recurrent stroke each year [3]. Although currently recommended therapies such as pharmacological thrombolysis, endovascular intervention, and rehabilitative strategies have proven to be beneficial, many stroke patients continue to have residual deficits despite treatment [4]. Regenerative therapy in the form of stem cells (neural stem cells, hematopoietic stem cells, and mesenchymal cells) is emerging as a promising treatment strategy to prevent stroke-related tissue damage, promote repair of damaged tissues and enhance functional recovery [5,6].

Stem cells (SCs) work through the mechanisms that involve integration into the host brain to replace within the damaged host tissue, neuroprotection involving downregulation of inflammatory and immune response, inhibiting apoptosis in a transplanted host as well as increasing endogenous repair process via vascular regeneration, induction of host brain plasticity, and recruitment of endogenous progenitors [6]. As stroke involves loss of multiple cell types including blood vessels, astrocytes, neurons, and oligodendrocytes, the neuroprotective and restorative property of stem cell-based therapy is indicative of a promising future in the treatment of stroke [7]. Because of similar pathophysiology and treatment strategy including thrombolysis in myocardial infarction (MI) and stroke, we can also use the cell-based therapy experience in MI as a guide to use in stroke management [7].

There have been multiple preclinical and clinical studies to establish the safety and efficacy of the use of SCs in stroke patients. Although many preclinical studies have reported a promising outcome of SC therapy, complete success has not been established through human clinical trials [5]. While some of the trials in humans have reported neutral outcomes and minor adverse effects related to the treatment, most of them have indicated that SCs are safe, and improve the functional outcome. However, there are multiple factors that may correlate with the safety and efficacy of the SCs including the host factors, type and source of SCs, dose, and route of delivery, time from stroke, and measures of safety and outcome [5].

Sufficient evidence to establish the safety and efficacy of the SC therapy in ischemic stroke is still lacking. It is urgent to explore the outcome of the therapy and its correlates to assess the possibility of bringing it to the clinics in the near future. Although several clinical trials of SC therapy in ischemic stroke conducted in the past have been reviewed, we could not find reviews of the studies conducted in recent years. The aim of this study is to highlight the findings of recent clinical trials published in the last six years to further establish the safety and efficacy of the SC therapies in patients with ischemic stroke and inform future research.

## Review

Methods and materials

Search Strategy

We manually searched for the clinical trials published from in between 2014 and 2020 using the PubMed database with the search strategy: “Stem cells or Stem cell therapy” and “Stroke or Middle cerebral artery or MCA or anterior cerebral artery or ACA or ischemic stroke” and “human” and “clinical trial”. We also searched http://clinicaltrials.gov to gain some additional required information about the studies.

Eligibility Criteria

We included the human clinical trials (controlled and noncontrolled, randomized and nonrandomized, single centered and multicentered, open-label and blind, phase I and II) published in English in between January 2014 and January 2020, that examined the safety and/or efficacy of SCs administered to patients with acute or chronic ischemic stroke via any routes (intravenous, intra-arterial, intrathecal, direct transplantation). Trials using any type of stem cells (mesenchymal, bone marrow-derived, umbilical cord-derived, neural, hematopoietic) with or without manipulation were included. Patients with hemorrhagic stroke and preclinical studies were excluded.

Data Extraction

We extracted data including date of publication, study design, location, number of participants in each trial, baseline inclusion criteria (at least one), the demographic profile of participants (gender, mean age), stem cell type, dose, route and time from onset of stroke to delivery, stroke type, and outcome: adverse events and efficacy. Measures of outcome measured latest were included if they were reported for >1 time points. In the case of controlled trials, the baseline characteristics of controls were similar to that of the treatment group, so we reported the baseline data only for the treatment group. Outcome measures for the treatment group were reported in comparison with that of controls. If any additional treatment was provided along with stem cell therapy, only stem cell therapy was considered. Two intervention groups (excluding the control group) in the same study, were reported separately. We extracted quantitative data from all available sources in each paper, including text and figures. Whenever this was not possible for instance, in the case of small graphs, we reported only the qualitative data. We developed a data abstraction spreadsheet using Microsoft Excel version 2016 (Microsoft Corp., Redmond, WA, USA) to organize the data. Two authors independently did the quality check of the selected clinical trials using the Physiotherapy Evidence Database (PEDro) scale and any difference in assessment was brought into concordance by discussing with the third researcher.

Results

**Figure 1:**
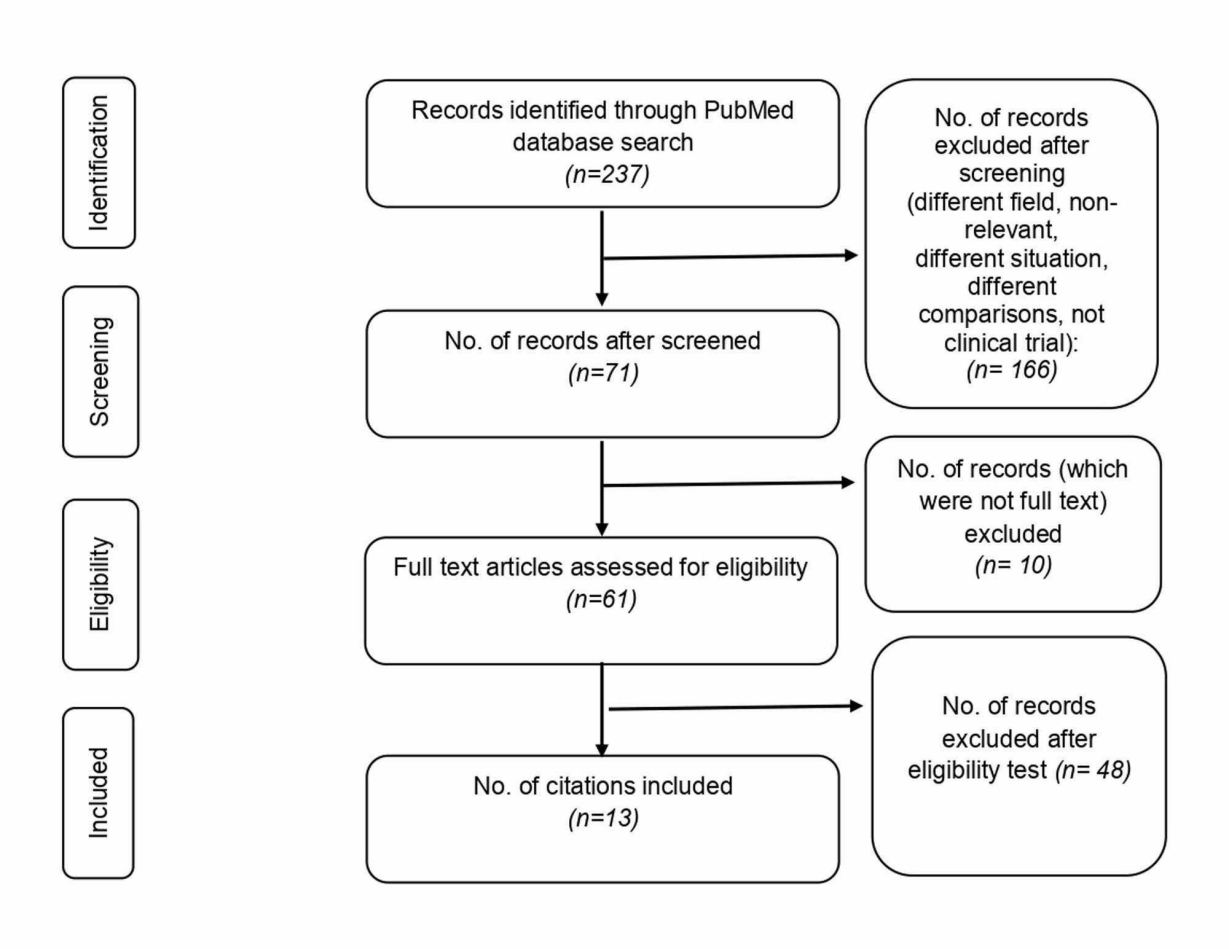
Selection process

We identified 237 articles from the database search. Screening by reading the abstracts resulted in the inclusion of 71 studies. Exclusion was because of different fields of study, different situations, non-relevance, and different study designs. We found full text for only 61 articles. Thirteen articles [8-20] that met the inclusion criteria were finally selected for review. The selection process is detailed in Figure 1. Seven [9,11,12,14,16,17,20] out of thirteen studies had a control arm, one of which was a historical control. Quality check of the studies by PEDro scale (Table 1) revealed the scores of the included studies which ranged from four to 10 (median: 5.5 [interquartile range (IQ) range: 3]). No study was excluded after quality assessment. 

**Table 1:** Quality check by PEDro scale PEDro: Physiotherapy Evidence Database

Criterion	Eligibility	Randomization	Concealed allocation	Baseline similarity	Subject blinding	Therapist blinding	Blinded assessment	85%of subjects assessed	Intention to treat	Statistical comparison	Point measure and measure of variability	TOTAL
Levy 2019 [8]	1	0	0	1	0	0	0	1	0	0	1	4
Savitz 2019 [9]	1	1	1	1	1	0	1	1	1	1	1	10
Laskowitz 2018 [10]	1	0	0	1	0	0	0	1	1	0	1	5
Bhatia 2018 [11]	1	1	0	1	0	0	1	1	1	1	1	8
Hess 2017 [12]	1	1	0	1	1	0	1	1	1	1	1	9
Kalladka 2016 [13]	1	0	0	1	0	0	0	1	1	0	1	5
Sprigg 2016 [14]	1	1	1	1	0	0	1	1	0	1	1	8
Steinberg 2016 [15]	1	0	0	1	0	0	0	1	1	0	1	5
Taguchi 2015 [16]	1	0	0	1	0	0	0	1	1	1	1	6
Prasad 2014 [17]	1	1	1	1	0	0	1	1	0	1	1	8
Qiao 2014 [18]	1	0	0	0	0	0	0	1	0	1	1	4
Qiao 2014 [18]	1	0	0	0	0	0	0	1	0	1	1	4
Banerjee 2014 [19]	1	0	0	1	0	0	0	1	1	0	1	5
Chen 2014 [20]	1	1	0	1	0	0	1	1	1	1	1	8

Study Characteristics

In the case of controlled clinical trials, we included characteristics of only the intervention group (wherever not specified), as the baseline characteristics of controls were found to be comparable to the intervention group. The baseline characteristics of the study participants are summarized in Table 2. Details of the type, dose, route, and time of delivery of stem cells along with the nature of the stroke are summarized in Table 3. 

**Table 2:** Baseline Characteristics M: Male, F: Female, ITT: Intention-to-treat, SD: Standard Deviation, IQR: Inter Quartile Range, NIHSS: National Institutes of Health Stroke Scale, mRS: modified Rankin Score, BI: Barthel Index, ESS: European Stroke Scale, NA: Not available

First Author Year	Study site	Study Design	Baseline Neurological Status	No. of participants (Control)	Age	Gender (M/F)
Levy 2019 [8]	US	phase I/II multi-center, open-label	mean NIHSS (range): 8 [6.5 to 10] mean BI±SD: 65±29 mean Geriatric depression scale score ±SD: 5.1±3.5	36	Mean (range) 61.1 [39–84]	27/9
Savitz 2019 [9]	US	Phase II randomized, sham-controlled, parallel-group, multicenter blinded assessments, ITT	mean NIHSS: 11 mRS ≥3.	29(19)	Mean (SD): 59.3 (10.03)	20/9
Laskowitz 2018 [10]	US	phase I, multisite, open‐label, prospective	Median NIHSS: 11 Median mRS: 4 Median BI: 17.4	10	Median(range): 65.5 (45–79)	10 M
Bhatia 2018 [11]	India	Phase II prospective, randomized, controlled, open-label, blinded-end point	NIHSS: >7, mean:10.6	10(10)	Mean ±SD: 57 ± 12.2	8/2
Hess 2017 [12]	US/UK	phase II, randomized, double-blind, placebo-controlled, dose-escalation trial	NIHSS: 8 to 20, mean (SD): 13·4 (3·6)	65(61)	Mean (SD): 61·8 (11·4)	35/30
Kalladka 2016 [13]	UK	Phase I open-label, single-site, dose-escalation study	median (IQR): NIHSS: 7(6–8) mRS: 3(3–4) BI: 12(11–14)	11	Median (IQR): 68 (61–75)	NA
Sprigg 2016 [14]	UK	single centered, pilot 2 x 2 factorial randomized (1:1) placebo-controlled trial, prospective, blinded outcome	NIHSS (mean (SD)): 6.7(4.7) mRS: >1	30(30)	Mean (SD): 66.8 (8.4)	17/13
Steinberg 2016 [15]	US	Phase I/IIa 2-year, open-label, single-arm	mean (SD): NIHSS 9.44 (1.89) mRS: 3.22 (0.43) ESS 58.44 (6.27) Mean stroke volume: 42cm3.	18	Mean (SD) 61.3 (10.29)	7/11
Taguchi 2015 [16]	Japan	Phase I/IIa nonrandomized open-label study design with historical control	mean ± SD: NIHSS:16.6 ± 4.7	12(59)	Mean ± SD: 67.4 ± 5.4	11/1
Prasad 2014 [17]	India	phase II, multicenter, parallel-group, randomized trial with blinded outcome assessment	Median (IQR): NIHSS 11 (6) BI: 25 (28.7)	60(60)	Mean ±SD: 50.7±11.6	41/19
Qiao 2014 [18]	China	Nonrandomized, open-label single centered single-arm clinical trial	Mean: NIHSS: 15.5 mRS: 5 BI: 5	2	Mean: 83	2 F
Qiao 2014 [18]	China	Nonrandomized, open-label single centered single arm	Mean: NIHSS: 4.5 mRS: 4.25 BI: 58.75	4	Mean: 42.75	3/1
Banerjee 2014 [19]	UK	Phase I prospective, nonrandomized, open-label	NIHSS: ≥8	5	Mean: 58.2	3/2
Chen 2014 [20]	Taiwan	Phase II randomized, single blind controlled	NIHSS: 9 to 20	15(15)	Mean (range): 50.1 (42-66)	12/3

**Table 3:** Nature of stroke and intervention MSC: Mesenchymal Stem Cells, ALD: Aldehyde dehydrogenase, HLA: Human Leukocyte Antigen, MCA: Middle cerebral artery, PCA: Posterior cerebral artery, ICA: Internal carotid artery, ACA: Anterior cerebral artery, LSA: Lenticulo striate artery, G-CSF: Granulocyte colony stimulating factor, PBSC: Peripheral blood stem cell, NSPC: Neural stem progenitor cell, UCB: Umbilical cord blood, BM: Bone marrow, hNSC: haemopoietic neural stem cell, IV: intravenous, IA: intra-arterial, TNCC: Total nucleated cell count, SC: sub-cutaneous

First Author Year	Stroke type and time of intervention	Type of stem cells	Route and Dose (m=million)
Levy 2019 [8]	> 6 months of ischemic stroke	Allogenic MSCs	IV/ 0.5-1.5 m cells/kg
Savitz 2019 [9]	11-17 days after MCA cortical and non-cortical ischemic stroke	autologous BM-derived ALD-401	IA(ICA)/ mean 3.08 m cells
Laskowitz 2018 [10]	3-9 days after MCA cortical ischemic stroke	Non-HLA matched, ABO matched, unrelated allogeneic UCB	IV/ single dose of 8.3 to 33.4 m TNCC/kg UCB
Bhatia 2018 [11]	8-15 days after MCA ischemic stroke	BM–derived mononuclear cells	IA/ maximum of 500 m cells
Hess 2017 [12]	24- 48 hours after hemispheric cortical ACA infarct	multipotent adult progenitor cells	IV/ (400 or 1200 m cells cells)
Kalladka 2016 [13]	Median (IQR) of 32 (14–44) months after MCA, ACA, or PCA cortical and sub cortical ischemic stroke	allogeneic immortalized human neural stem-cell	Intracerebral implantation/ single doses of 2, 5, 10, or 20 m hNSCs
Sprigg 2016 [14]	Mean(range) of 22.0 (7–36) months post TACS, PACS, LACS, TIA	G-CSF	SC/ Filgrastim, 1 m iu/kg
Steinberg 2016 [15]	Mean (range) of 22.0 (7–36) months after MCA or LSA cortical or subcortical ischemic stroke	modified BM-derived MSCs	Surgical transplantation/ 2.5, 5.0, or 10 m SB623 cells.
Taguchi 2015 [16]	7-10 days after MCA, ICA, PCA, ACA stroke	Autologous BM mononuclear cells	IV/ 25000 and 34000 m cells
Prasad 2014 [17]	Median (IQR) of 18.5(9.2) days after ischemic stroke	autologous BM Stem Cells	IV/ 280.75 m mononuclear cells containing 2.9 m CD34+ cells
Qiao 2014 [18]	Mean of 2.5 months after ACA, MCA ischemic stroke	MSCs derived from UC	IV/ MSCs (0.5 m/kg weight) 4 doses
Qiao 2014 [18]	Mean of 9.25 months after ACA, MCA ischemic stroke	MSCs+ NSPCs	IV MSCs (0.5 m/kg followed by implantation in cerebellomedullary cistern: MSCs (5 m/patient) and NSPCs (6 m/patient)
Banerjee 2014 [19]	< 7 days of severe ACA ischemic stroke	autologous, immunoselected CD34+ stem/progenitor cell	IA/ 1.2-2.79 m cells
Chen 2014 [20]	Mean ±SD (range) of 2.7±1.4(0.6-5) years after MCA ischemic stroke	G-CSF and CD34(+) immunosorted PBSCs	SC G-CSF injections (15 µg/kg/day) and Implanted 3-8 m CD34(+) immunosorted PBSCs

Study Outcome

The outcomes (adverse events and efficacy) of the treatment reported in the included studies are summarized in Table 4. For controlled studies, the outcome measures for the treatment group are detailed in comparison with that of controls. Serious adverse events reported were transient ischemic attack, seizure, asymptomatic subdural hematoma/hygroma, urinary tract infecton (UTI), sepsis, pneumonia, hyperglycemia, neutrophilia, shingles, ischemic stroke, cellulitis, muscle cramps, fracture neck femur, and peripheral vascular disease. None of these events were attributed to the cell therapy but some were reported probably to be procedure-related. 

**Table 4:** Outcome- Adverse events and Efficacy CAD: Coronary artery disease, GCSF: Granulocyte Colony Stimulating Factor, PBSC: Peripheral blood stem cell, NIHSS: National Institute of Health Stroke Scale, BI: Barthel Index, mRS: modified Rankin Score, SD: Standard deviation, IQR: Inter-quartile range, EQ-5D QoL: Euro Quality of Life 5-Dimension Questionnaire, ESS: European Stroke Scale, EMS: ESS Motor Subscale, BMSC: Bone marrow-derived stem cell

First Author Year	Adverse events (AE)	Efficacy
Levy 2019 [8]	2 died (CAD) and 15 serious AE (all unrelated to therapy): infections, vascular disorders, and pain syndromes.	At 12 months: Significant gain in all behavioral end points: BI increased by 10.8±15.5 points (P<0.001); the proportion of patients achieving excellent functional outcome (BI ≥95) increased from 11.4% at baseline to 35.5%. NIHSS decreased by −2 [−3.5 to −0.5]. Geriatric depression scale score changed by −1.4±3.8.
Savitz 2019 [9]	No serious treatment related AE except for seizures (frequency 4 times more in the treatment group, so probably treatment related)	At 1 year: no statistically significant differences in mean mRS, BI, NIHSS. No correlation between dose of administration and mRS outcome
Laskowitz 2018 [10]	Only one AE (pruritis of moderate severity) possibly related to the investigational treatment.	At 3 months: All participants improved by at least 1 grade in mRS (mean ±SD 2.8 ± 0.9) and by at least 4 points in NIHSS (mean 5.9 ± 1.4), relative to baseline. BI increased by mean ±SD 52.0 ± 24.7; range 10–80
Bhatia 2018 [11]	No procedure-related complication was seen in any patient. Mortality: 2(Control)/1(treatment) (0.53); new infarct in 1(treatment) (0.305); all insignificant	At 6 months: Good clinical outcome (mRS score < 2) in 80% patients in the treatment group vs 40% in the control group (p=0.068). Improvement in the mRS in both groups but, statistically significant only in the treatment group (P = .009). Significant improvement in the BI only in the intervention group (P =. 004)
Hess 2017 [12]	Life-threatening adverse events or death: 8 (12%) treatment vs 15 (25%) control. Secondary infections :25 (39%) treatment vs 29 (48%) control	At 1 year: mRS ≤2 achieved in 51% of treatment group vs 44% of control; NIHSS improvement of ≥75% in 49% of treatment group vs 46% of control; Excellent outcome achieved in 23% of treatment vs 8% of control (p= 0·0206) [Excellent outcome: composite of mRS ≤1, NIHSS ≤1, and BI ≥95]
Kalladka 2016 [13]	1 new ischemic stroke (not treatment relate superficial malignant melanoma occurred in 1 patient with a history of chronic sun exposure.	At 2 years: improvement in NIHSS score by a median of 2 points (range: 0- 5). At 12 months: Patient-reported overall health status improved by a median of 18 points (IQR –5 to 30)
Sprigg 2016 [14]	More patients in the treatment group (9) reported serious adverse events than in the control group (3), although not statistically significant (p = 0.10)	At 1 year: No significant difference in dependency or disability outcomes between the treatment and control groups. At 3 months: Significant improvement in EQ-5D QoL in the treatment group (+0.15) vs control (-0.02)
Steinberg 2016 [15]	At least 1 treatment-emergent adverse event. 6 patients experienced 6 serious adverse events (2 were probably or definitely related to surgical procedure; none were related to cell treatment). No dose-limiting toxicities or deaths	At 12 months: significant improvement in: (1) ESS: mean increase 6.88 (95% CI, 3.5-10.3; P<0.001), (2) NIHSS: mean decrease 2.00 (95% CI, -2.7 to -1.3; P<0.001), (3) Fugl-Meyer total score: mean increase 19.20 (95% CI, 11.4-27.0; P<0.001), and (4) Fugl-Meyer motor function total score: mean increase 11.40 (95% CI, 4.6-18.2; P<0.001). No change in mRS
Taguchi 2015 [16]	1 person: recurrent stroke. Otherwise, no apparent adverse effects of administering bone marrow cells were observed.	At 1 month: Mean improvement in NIHSS score was 4.8 ± 4.6 (P
Prasad 2014 [17]	No serious adverse events: others were similar in the 2 arms.	At 6 months: No significant difference between BMSCs arm and control arm in the BI (63.1 versus 63.6; P=0.92), mRS shift analysis (P=0.53) or score >3 (47.5% versus 49.2%; P=0.85), NIHSS score (6.3 versus 7.0; P=0.53), change in infarct volume (-11.1 versus -7.36; P=0.63).
Qiao 2014 [18]	Low fever in 6 cases that usually lasted 2-4 days after each therapy. There was no evidence of neurological deterioration or neurological infection. No tumorigenesis was found at a 2-year follow-up.	At 2 years: Improved neurological functions (NIHSS), disability levels (mRS), and daily living abilities (BI).
Banerjee 2014 [19]	No significant treatment-related adverse effects. One patient developed renal dysfunction 2 weeks after the infusion and subsequently experienced an episode of pneumonia.	At 6 months: Improvements in clinical functional scores (mRS and NIHSS) and reductions in lesion volume
Chen 2014 [20]	No deaths, serious AEs, or other unfavorable symptoms in the follow-up period after G-CSF treatment in the PBSC group or the control group.	At 12 months: Improvement in NIIHS grade 9.3 ± 0.5 to grade 5.5 ± 1.8, ESS grade 69.3 ± 7.8 to grade 76.1 ± 8.1, EMS grade 23.9 ± 8.2 to grade 30.5 ± 8.8, mRS grade 2.9 ± 0.3 to grade 2.1 ± 0.3 in the PBSC group (were significantly greater )compared to control.

Discussion

Cell-based therapy offers a promising future in the field of ischemic stroke management amidst the limited benefits seen with the existing therapies such as thrombolysis and endovascular intervention [4]. Several factors may come into play when we analyze the safety and efficacy of cell-based therapies, such as the type of stem cells, modifications, dose, and route of delivery along with the patient’s baseline characteristics and time of intervention. In this study, we attempted to review the results of the clinical trials of stem cell treatments conducted in the last six years in patients with ischemic stroke regarding the safety and efficacy of the therapy.

In our review, we included thirteen clinical trials [8-20] conducted from 2014 to 2020, which used stem cells to treat ischemic stroke patients. Seven studies [9,11,12,14,16,17,20] out of 13 were randomized controlled trials (RCT) and six [8,10,13,15,18,19] were single-arm studies. Only three RCTs, Savitz et al, Sprigg et al., and Prasad et al. of the seven showed no significant change in the outcome measures [9,14,17]. These studies had a relatively higher methodological quality and used allocation concealment and blinded outcome. As allocation concealment helps to minimize selection bias, the rest of the studies which showed the positive results without allocation concealment might have exaggerated the effects of treatment [21]. In Prasad et al. [17] and Savitz et al. [9], intervention was not in the acute stage, and the baseline National Institutes of Health Stroke Scale (NIHSS) were higher (which means poor prognosis), which might have caused to yield no benefits. Prasad et al. also reported that future trials should focus on treating stroke within one week of onset [17]. In Sprigg et al., relatively non-serious and chronic cases of stroke were enrolled, which might have caused to miss the noticeable changes with the therapy [14]. The chance of type 2 error due to small sample sizes is also possible.

It is noteworthy that four RCTs, Bhatia et al., Hess et al., Taguchi et al., and Chen et al. out of seven RCTs, which also had higher methodological quality on the basis of study design, outcome measurement and analyses, revealed positive results [11,12,16,20]. In Hess et al. [12], Taguchi et al. [16], and Bhatia et al. [11], relatively early intervention (at 24 hours, 7-10 days, and 8-19 days respectively) using intravascular delivery (intravenous or intraarterial) in the patients with higher baseline NIHSS (13.5, 16.5, 10.6 respectively) yielded positive results. This is in accordance with the previous findings that intravascular delivery of stem cells may be ideal within 24 hours - a month of the onset of stroke [22,23,24]. For the cases of stroke after months to years of onset, direct implantation of cells can be the choice as it permits better engraftment after the initial inflammatory response is over [25]. Accordingly, we found that in Chen et al., treatment with subcutaneous (s.c.) injection of Granulocyte Colony Stimulating Factor (GCSF) and intracerebral implantation of CD34(+) immunosorted Peripheral Blood Stem Cells (PBSCs), as late as six months to five years of stroke onset in patients with baseline NIHSS of 8-20, demonstrated positive results [20]. Especially in Taguchi et al., an important thing to account is the dose-response relationship of the SCs and efficacy outcomes in the patients, which explains strong evidence of the causal relationship between the therapy and response [16]. In contrast, no correlation between the dose of administration and modified Rankin Scale (mRS) outcome was seen in Savitz et al. [9]. However, analysis of the dose-response relationship of the SCs and efficacy outcomes in the future cannot be emphasized more to determine the measure of benefits of the treatment.

Although all of the single-arm studies [8,10,13,15,18,19] showed positive results, there is a chance that the natural progression of the disease has some role, if not all, on improvement of the patients. These studies possibly gave higher estimates of neurological improvement, and publication bias is also possible. There is a chance of type I error as most of them were found lacking effective study design such as randomization, blinding, allocation concealment, intention-to-treat (ITT) analysis, and statistical comparison. However, these studies can be useful to look out for the adverse events associated with cell therapy.

Regarding the route of administration, although intra-arterial (i.a.) is preferred to intravenous (i.v.) route because of no first-pass metabolism in i.a. route, six out of seven studies in our review, which used i.v. route correlated with positive results, except Prasad et al. [17,26]. This is similar to the studies in rodents which yielded similar or greater benefits associated with i.v. route than through i.a. or intracerebral route [27,28]. Six trials that used other routes (i.a., s.c. or direct implantation) showed mixed outcomes (four showed positive results and two showed neutral results) but more procedural adverse effects (such as hematomas, infections) were reported in most of them. For instance, in Steinberg et al. which used the surgical transplantation method, post-surgery headache, a subdural fluid collection and a life-threatening seizure were reported probably or definitely related to the procedure [15]. Also, in Savitz et al., i.a. route correlated with no benefits, rather some procedural adverse effects were seen [9].

Among the adverse events reported in the included clinical trials, only minor or less severe adverse events were due to the stem cell treatment as such. The serious adverse events reported, such as transient ischemic attack, seizure, asymptomatic subdural hematoma/hygroma, urinary tract infection (UTI), sepsis, pneumonia, hyperglycemia, neutrophilia, shingles, ischemic stroke, cellulitis, muscle cramps, fracture neck femur, and peripheral vascular disease were not attributed to the cell therapy although some were reported probably to be procedure-related. Only two studies followed up the participants up to two years of treatment for the outcome and the rest of the studies followed up only up to one year of treatment or less than that. To reject the potential of adverse events especially tumorigenesis, a long-term follow-up is needed [29]. However, as we included the trials using any type of stem cells in our review, adverse events specific to stem cell type could not be effectively interpreted. Direct comparison of the source of stem cells would have helped to establish the ideal source and delivery technique and to make the outcome measures more comparable. Overall, as no treatment-related mortality, tumorigenesis, or major adverse effects were reported, this encourages us to proceed with the further stages of trials in a larger scale.

There is no established mechanism of action of the stem cells and a specific intention of treatment has not been recognized yet [30,31]. Accurate hypotheses governing those factors including type of stem cells, techniques of extraction, modification, and delivery is deemed necessary in the future. Long term follow-up to watch for the potential adverse events and monitor the effectiveness of the therapy including the survival benefit using survival analysis seems important to define the prognostic indices after the therapy. Outcome measures also need to be more specific to the deficit in a patient, for instance, one with aphasia should be assessed for aphasia rather than a general neurological assessment as proposed by Cramer et al. to use “modality-specific outcome measures” [32]. Clinical trials combining stem cell treatment with traditionally established treatment such as thrombolysis, endovascular intervention, and physiotherapy should be conducted in a large scale to explore the potential benefits. None of the reviewed studies included patient-focused outcomes, although they used the objective scales to measure impairment and functional status (i.e., NIHSS, mRS, or BI).

Our study has some limitations. We excluded the studies published in languages other than English, which might have limited our comprehensiveness. Although we did an extensive search of the relevant clinical trials, there is a chance that we might have missed some. We could not do statistical analysis of the measured outcome; hence quantitative comparisons could not be done between the studies. Because of difficulty to accurately extract the data from small graphs and figures, we mentioned only the reported qualitative data for some studies, and we did not contact the author. Overall, the sample size of the included studies was relatively small. While Hess conducted the largest study with 125 subjects (60 in the control arm and 65 in the treatment arm), in a blinded fashion, Banerjee conducted with as less as five participants as phase I, nonrandomized, open-label trial [12,19].

## Conclusions

The clinical trials we included in our review showed mixed results in terms of efficacy of the SCs in the treatment of ischemic stroke while assessment of safety profile yielded promising results as there were only minor side effects related to the cell therapy. There are discrepancies and heterogenicity in results to date in this emerging field which reflect the obstacles we must overcome to bring the SCs to the bedside. Before the stem cell therapy reaches the clinic, there needs to be larger, adequately powered, and well-designed clinical trials with more comparable results to assess and establish the safety and efficacy of SCs in stroke patients.
